# Inferior vena caval thrombosis complicating pyogenic liver abscess after pancreatoduodenectomy: a case report

**DOI:** 10.1186/s40792-015-0080-y

**Published:** 2015-09-08

**Authors:** Hidemasa Kubo, Fumihiro Taniguchi, Katsumi Shimomura, Kenji Nanishi, Yasuo Ueshima, Akiyuki Takahashi, Yasuhiro Shioaki, Eigo Otsuji

**Affiliations:** Department of Surgery, Japanese Red Cross Kyoto Daiichi Hospital, 15-749, Hommachi, Higashiyama-ku, Kyoto-shi, Kyoto, Japan; Department of Respiratory Surgery, Japanese Red Cross Kyoto Daiichi Hospital, 15-749, Hommachi, Higashiyama-ku, Kyoto-shi, Kyoto, Japan; Department of Cardiovascular Surgery, Japanese Red Cross Kyoto Daiichi Hospital, 15-749, Hommachi, Higashiyama-ku, Kyoto-shi, Kyoto, Japan; Division of Digestive Surgery, Department of Surgery, Kyoto Prefectural University of Medicine, 465 Kajii-cho, Kawaramachihirokoji, Kamigyo-ku, Kyoto-shi, Kyoto, Japan

**Keywords:** Inferior vena caval thrombosis, Pyogenic liver abscess, Thrombectomy

## Abstract

Pyogenic liver abscess (PLA) complicated by inferior vena caval (IVC) thrombosis is rare but life-threatening. We experienced a case of PLA complicated by an IVC thrombus close to the right atrium after pancreatoduodenectomy. A 75-year-old man had undergone pancreatoduodenectomy with modified-Child reconstruction for pancreatic cancer 3 years prior, and no recurrence was noted on follow-up. He was admitted to our hospital owing to fever and general fatigue. PLA and septic shock were diagnosed, and conservative therapy with antibiotics was initiated. His general condition gradually improved, but a thrombus in the middle hepatic vein and IVC was noted on follow-up computed tomography on hospital day 8. Although anticoagulant therapy using heparin was started, the thrombus size increase and extended to the right atrium. Considering the risk of pulmonary embolism, we planned a surgical intervention with a cardiovascular surgeon to remove the thrombus. During surgery, we made an incision in the right atrium and removed the thrombus using extracorporeal circulation. After removal, we dissected the middle hepatic vein using an automated suturing device to prevent the thrombus from extending into the IVC. The patient was discharged 10 weeks after surgery. Eighteen months post-intervention, there was no recurrence of either PLA or thrombi. Our experience suggests that physicians should consider the existence of a middle hepatic vein and IVC thrombi when examining PLA patients and that surgical intervention can be applied successfully in such cases.

## Background

With increasingly effective diagnostic tools and treatment modalities available, the prognosis of patients with pyogenic liver abscess (PLA) has improved. However, PLA remains a life-threatening disease with reported mortality rates of 11–31 % [1, 2]. Although PLA can be accompanied by a number of complications, such as abscess rupture and metastatic central nervous system infections [3, 4], inferior vena caval (IVC) thrombosis is rare. IVC thrombosis can cause pulmonary embolism. Thus far, there has been only one report of an IVC thrombus complicating PLA [5]; therefore, the most suitable and prompt management approach has yet to be established. Here, we present a case of PLA complicated by an IVC thrombus close to the right atrium that developed after pancreatoduodenectomy that was successfully treated surgically.

## Case presentation

### Case details

**Fig. 1:**
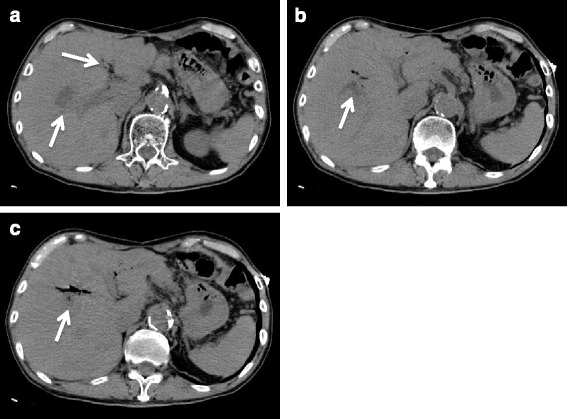
Computed tomography performed on admission. **a**, **b** Low-density area with air density detected in segment 8 of the liver (*arrow*). **c** The *arrow* shows air density in the middle hepatic vein. We determined that the abscess had ruptured into the middle hepatic vein

A 75-year-old man had undergone pancreatoduodenectomy with modified-Child reconstruction for pancreatic cancer 3 years earlier and did not experience recurrence on follow-up. He was admitted to our hospital owing to fever and general fatigue. His vital signs on admission were as follows: consciousness, alert; heart rate, 69 beats/min; blood pressure, 77/48 mmHg; respiratory rate, 15 breaths/min; peripheral capillary oxygen saturation, 95 % on room air; and body temperature, 36.5 °C. On physical examination, his abdomen was soft and flat, and his skin turgor was poor. We detected elevated inflammatory levels and decreased renal function. Non-contrast computed tomography (CT) revealed an approximately 30-mm low-density area and an air density in segment 8 of the liver (Fig. 1). Furthermore, there was an air density in the middle hepatic vein. We diagnosed PLA due to cholangitis in the middle hepatic vein and septic shock. We subsequently initiated conservative treatment using antibiotics (meropenem 1.5 g/d).

The patient had no medical history of any diseases, except pancreatic cancer. Laboratory results on admission were as follows: leukocyte count, 14.5 × 10^3^/μL; hemoglobin level, 10.9 g/dL; platelet count, 23 × 10^3^/μL; aspartate aminotransferase level, 68 IU/L; alanine aminotransferase level, 68 IU/L; alkaline phosphatase level, 747 IU/L; total protein level, 4.9 g/dL; total bilirubin level, 1.6 mg/dL; blood urea nitrogen level, 99 mg/dL; creatinine level, 2.71 mg/dL; amylase level, 43 IU/L; C-reactive protein level, 15.6 mg/dL; prothrombin percentage activity, 53 %; and activated partial thromboplastin time, 37.2 s.

**Fig. 2:**
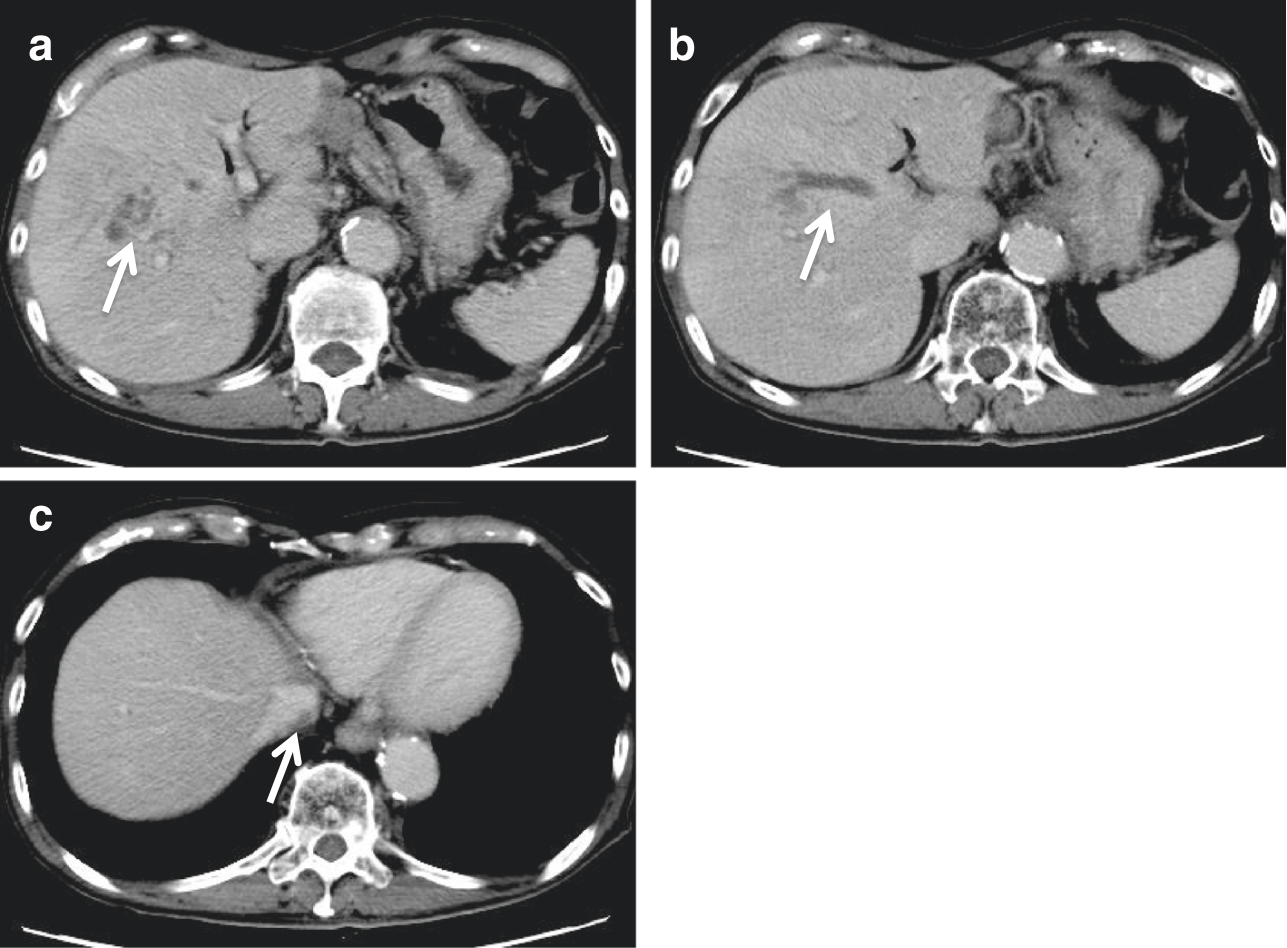
Follow-up computed tomography on hospital day 8. **a** The abscess cavity was reduced slightly (*arrow*). **b** We detected a thrombus in the middle hepatic vein (*arrow*). **c** The thrombus extended to the inferior vena cava (*arrow*)

**Fig. 3:**
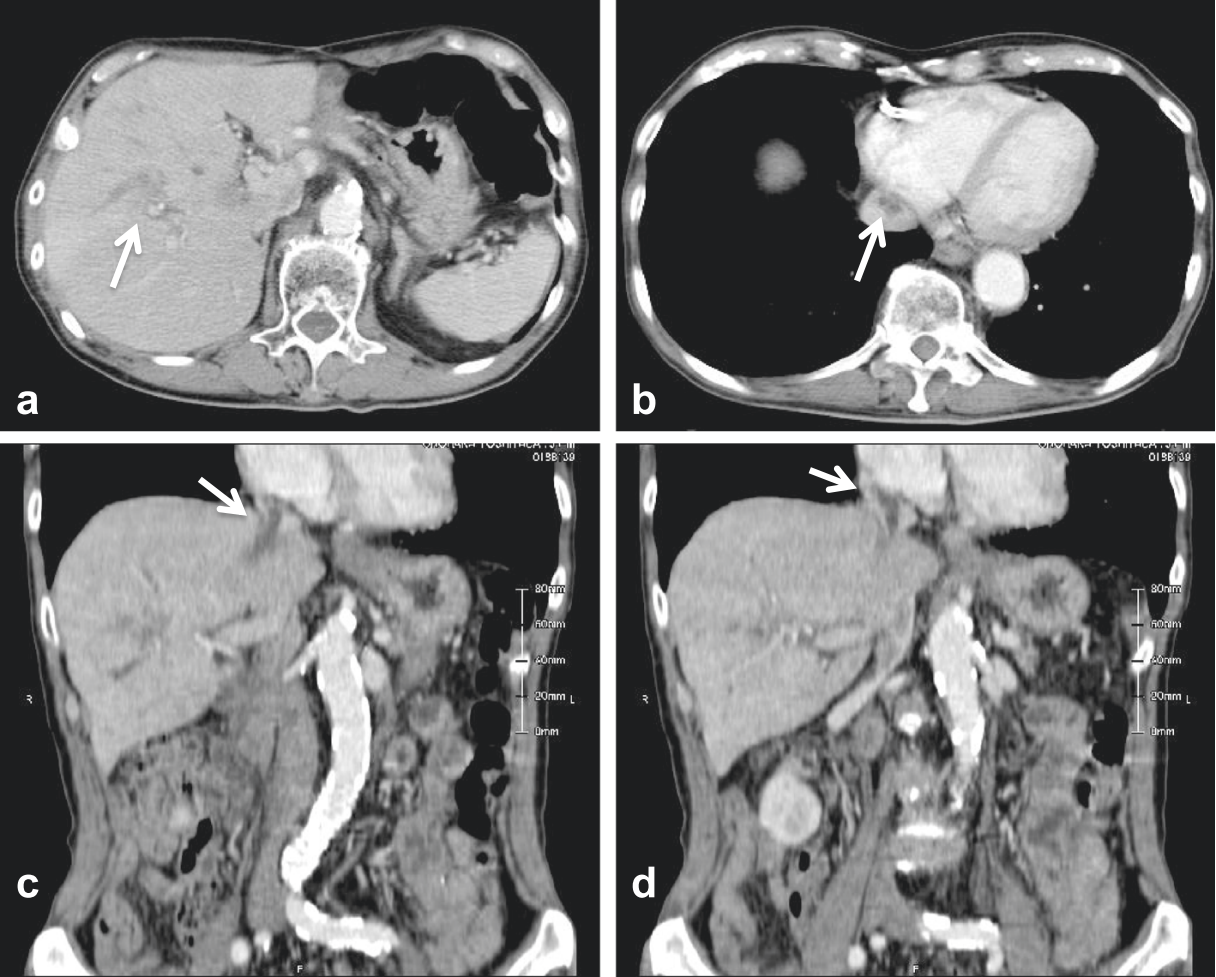
Follow-up computed tomography on hospital day 25. **a** The abscess was reduced in size (*arrow*) **b**, **c**, **d** The thrombus enlarged further and extended close to the right atrium

After being admitted to the hospital, the patient gradually recovered with antibiotic treatment. However, a contrast CT scan on hospital day 8 revealed a thrombus in the middle hepatic vein (Fig. 2). Therefore, we started anticoagulant therapy using heparin (15,000 U/d) and continued meropenem 1.5 g/d treatment. A follow-up CT scan on hospital day 17 revealed that the abscess had reduced in size but that the thrombus had slightly enlarged. We thought that the abscess was being controlled by the antibiotics; therefore, we continued the same treatment approach. A follow-up CT scan on hospital day 25 showed that the abscess had reduced further. However, the thrombus had enlarged again and reached the IVC close to the right atrium (Fig. 3). Considering the risk of pulmonary embolism, we planned a surgical intervention. A blood culture on admission was positive for *Citrobacter freundii*.

**Fig. 4:**
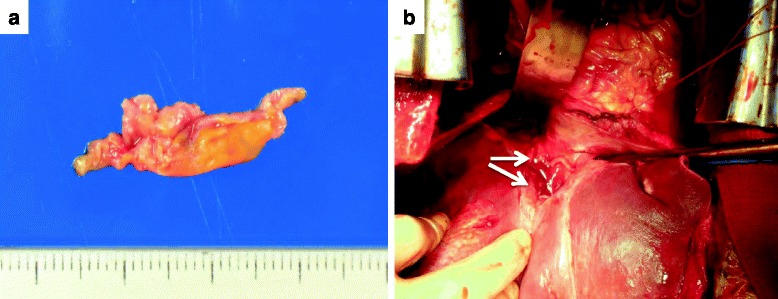
**a** The thrombus removed from the inferior vena cava was yellow. **b** We dissected the middle hepatic vein using an automated suturing device to prevent the thrombus from extending into the inferior vena cava (*arrows*)

The patient underwent a thrombectomy using extracorporeal circulation. We made an incision in the right atrium and removed the thrombus through the orifice of the middle hepatic vein. The thrombus was yellow (Fig. 4a), and pathological observation showed that it consisted of fibrous material including many neutrophils and macrophages. Accordingly, we made a diagnosis of septic thrombosis. Intraoperative trans-esophageal echocardiography showed a thrombus in the right pulmonary artery. A preoperative CT scan showed no thrombus in the right pulmonary artery; therefore, we believed that the embolism might have occurred since the preoperative CT scan was performed. We made an incision in the artery and attempted to remove it using a Fogarty catheter. We then noted extensive bleeding from the right bronchus. We diagnosed a fistula of the right pulmonary artery and bronchus caused by catheter handling. To control the bleeding, the patient underwent a right lower lobectomy. We did not identify the pulmonary thrombus. Subsequently, we transferred the operating field to the abdomen. We dissected the middle hepatic vein using an automated suturing device to prevent the thrombus from extending into the IVC (Fig. 4b). The operation took 14 h and 5 min, while the total blood loss was 11,920 g.

**Fig. 5:**
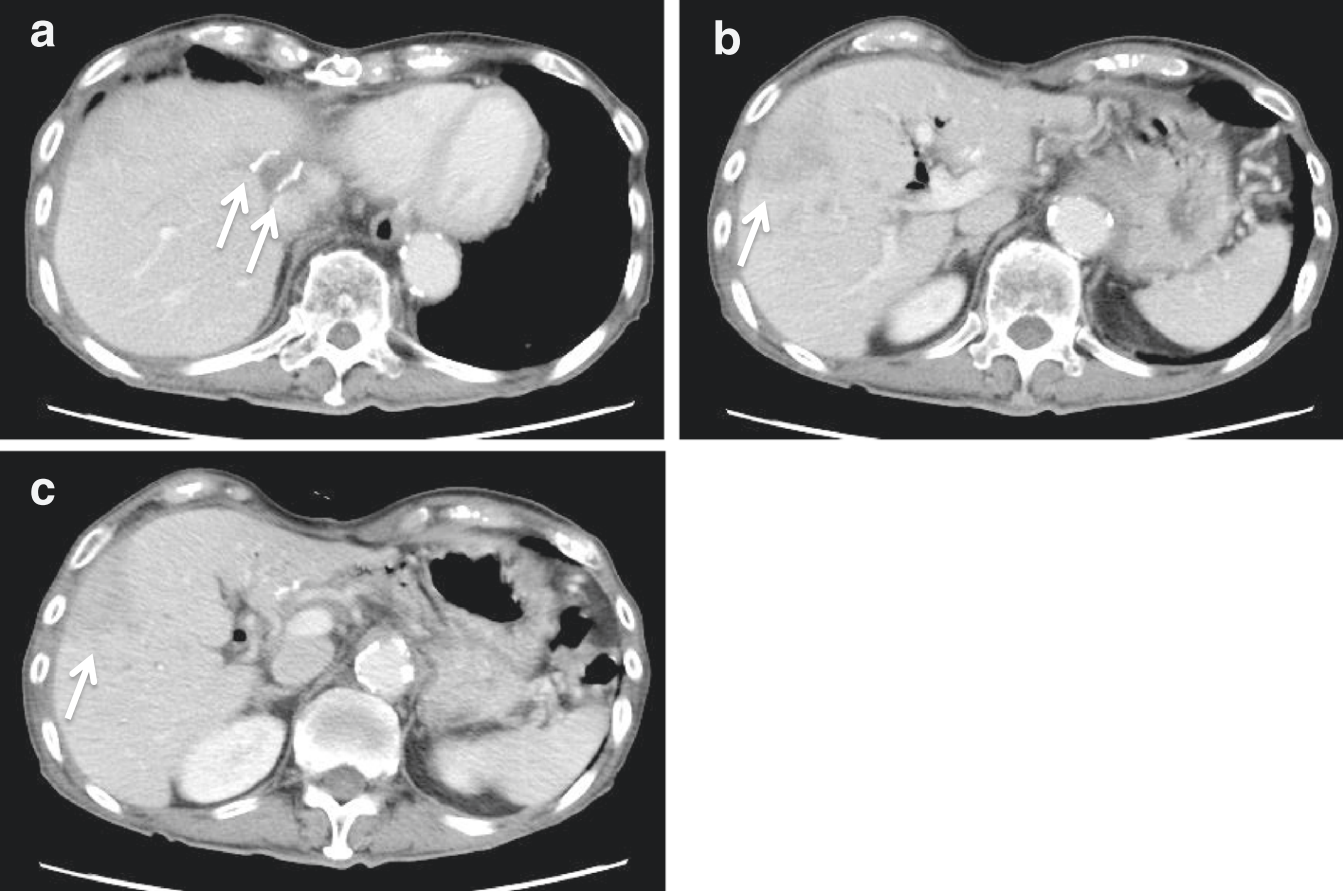
Follow-up computed tomography before discharge. **a** *Arrows* show the stapler that dissected the middle hepatic vein. **b**, **c** The abscess disappeared. No atrophic changes were seen in the drainage area of the middle hepatic vein, but the density of the area was lower than that of the other area because of the change in venous drainage (*arrow*)

The patient was discharged 10 weeks after surgery despite experiencing respiratory failure and undergoing tracheotomy. Before discharge, we confirmed the disappearance of the liver abscess on a CT scan. No atrophic changes were seen in the drainage area of the middle hepatic vein, but the density of the area was lower than that of the other owing to a change in venous drainage (Fig. 5). At the present, 18 months after the surgery, the patient experienced no recurrence of either the liver abscess or thrombus.

### Discussion

PLA reportedly occurs in 20–22 of every 100,000 hospitalized patients, and the mean age at the time of admission is reportedly 55.5–56.4 years [1, 2, 6]. PLA affects the sexes equally [1, 2], but one study reported that more men suffer from the disease than women (69.6 %) [6]. The infection route is classified as biliary, portal, hematogenous, direct extension, abdominal trauma, chronic granulomatous disease, or cryptogenic [1]. Fever, abdominal pain, and hepatomegaly comprise the PLA triad. PLA is treated with either broad-spectrum antibiotics and drainage or antibiotics alone [1, 2]. A number of complications have been reported in cases of PLA, such as abscess rupture and metastatic central nervous system infections [3, 4], but vascular complications such as IVC thrombosis are rare. A few cases have been reported for amebic liver abscess [7–11] compared to just one for PLA [5]. IVC thrombosis is a life-threatening complication because of the associated risk of pulmonary embolism.

Our case involved a thrombus in the middle hepatic vein and the IVC complicating PLA after pancreatoduodenectomy with hepaticojejunostomy. Cholangitis reportedly occurs in 6.4 % of patients after hepaticojejunostomy [12]. In our case, blood culture on admission yielded *C. freundii*, an intestinal bacterial species. There was no evidence of abdominal trauma, intestinal infection, or chronic granulomatous disease. Therefore, the PLA seemed to occur through the biliary route.

Virchow’s triad, which consists of abnormalities in blood composition, vessel wall components, and blood flow, is reportedly associated with thrombus formation [13]. Jun et al. [3] reported that spontaneous rupture of PLA occurs in 3.8 % of cases. Liver cirrhosis, gas-forming abscess, abscess ≥ 6 cm in diameter, and other septic metastases have been reported as risk factors for rupture. In the present case, a gas-forming abscess was detected by CT upon admission; this abscess ruptured affecting the middle hepatic vein. Direct injury of the endothelial cells of the middle hepatic vein might explain the thrombus formation in this case. Bagri et al. [5] reported that sepsis-induced endotheliitis seems to be the most important factor for thrombus formation. In the present case, we considered the endothelial condition (injury and inflammation) as the most important factor of thrombus formation as well as the dehydrated state of the patient on admission.

The optimal management of IVC thrombosis complicating PLA has not yet been established. Infection control and anticoagulation therapy might prevent thrombus occurrence or enlargement. However, a threatened pulmonary embolism such as right atrium thrombosis should be removed, regardless of whether the patient’s infection could be controlled. Bagri et al. [5] successfully used surgical intervention and antibiotics to treat IVC thrombosis complicating PLA following aspiration. In our case, we considered using an indwelling IVC filter to prevent a pulmonary embolism. We attempted to indwell the filter at the heart side from the hepatic vein, but it was too difficult to perform for technical reasons. Therefore, we attempted anticoagulation therapy. Although we started anticoagulation therapy as soon as possible, the thrombus extended close to the right atrium. We consulted a cardiovascular surgeon for treating the thrombus and chose surgical intervention. Because the thrombus enlarged despite infection control, we believe that the surgical timing was appropriate. The patient underwent surgical thrombectomy with extracorporeal circulation. With regard to the thrombus of the right pulmonary artery that was found intraoperatively, there was a reflection. We could not identify it, and it was diagnosed only on intraoperative trans-esophageal echocardiography, so there was a possibility of no existing in fact. However, because it was diagnosed intraoperatively, we attempted to remove the thrombus using a Fogarty catheter. We ultimately treat the patient but encountered severe complications such as fistula of the right pulmonary artery and bronchus that induced extensive bleeding. As such, we should use a Fogarty catheter more carefully.

In the case of IVC thrombosis complicating an amebic liver abscess, one case was reportedly treated successfully with conservative therapy such as oral metronidazole, abscess aspiration, and anticoagulation [7], whereas others were treated with surgical removal [8–11]. The effect of anticoagulation therapy is not yet clear for thrombi complicating PLA. The surgical stress induced by extracorporeal circulation is excessive, but a pulmonary embolism is life-threatening. Therefore, it is important to choose the most suitable surgical timing for IVC thrombosis complicating PLA.

## Conclusions

Here, we presented a case of IVC thrombosis close to the right atrium complicating PLA after pancreatoduodenectomy that was treated surgically. Physicians should consider the existence of a middle hepatic vein and IVC thrombosis when examining patients with PLA. Early diagnosis and prompt treatment are essential to reducing thrombosis-associated mortality.

## Consent

Informed written consent was obtained from the patient for the publication of this case report.

## Abbreviations

CT: computed tomography
IVC: inferior vena caval/inferior vena cava
PLA: pyogenic liver abscess
